# Optimization of l-malic acid production from acetate with *Aspergillus oryzae* DSM 1863 using a pH-coupled feeding strategy

**DOI:** 10.1186/s12934-022-01961-8

**Published:** 2022-11-23

**Authors:** Aline Kövilein, Vera Aschmann, Lena Zadravec, Katrin Ochsenreither

**Affiliations:** Institute of Process Engineering in Life Sciences 2-Technical Biology, Karlsruhe Institute of Technology (KIT), Fritz-Haber-Weg 4, 76131 Karlsruhe, Germany

**Keywords:** Malate, Organic acid, Acetic acid, Filamentous fungi, Feeding strategy, Product inhibition

## Abstract

**Background:**

Malic acid, a dicarboxylic acid mainly used in the food industry, is currently produced from fossil resources. The utilization of low-cost substrates derived from biomass could render microbial processes economic. Such feedstocks, like lignocellulosic hydrolysates or condensates of fast pyrolysis, can contain high concentrations of acetic acid. Acetate is a suitable substrate for l-malic acid production with the filamentous fungus *Aspergillus oryzae* DSM 1863, but concentrations obtained so far are low. An advantage of this carbon source is that it can be used for pH control and simultaneous substrate supply in the form of acetic acid. In this study, we therefore aimed to enhance l-malate production from acetate with *A. oryzae* by applying a pH-coupled feeding strategy.

**Results:**

In 2.5-L bioreactor fermentations, several feeding strategies were evaluated. Using a pH-coupled feed consisting of 10 M acetic acid, the malic acid concentration was increased about 5.3-fold compared to the batch process without pH control, resulting in a maximum titer of 29.53 ± 1.82 g/L after 264 h. However, it was not possible to keep both the pH and the substrate concentration constant during this fermentation. By using 10 M acetic acid set to a pH of 4.5, or with the repeated addition of NaOH, the substrate concentration could be maintained within a constant range, but these strategies did not prove beneficial as lower maximum titers and yields were obtained. Since cessation of malic acid production was observed in later fermentation stages despite carbon availability, a possible product inhibition was evaluated in shake flask cultivations. In these experiments, malate and succinate, which is a major by-product during malic acid production, were added at concentrations of up to 50 g/L, and it was found that *A. oryzae* is capable of organic acid production even at high product concentrations.

**Conclusions:**

This study demonstrates that a suitable feeding strategy is necessary for efficient malic acid production from acetate. It illustrates the potential of acetate as carbon source for microbial production of the organic acid and provides useful insights which can serve as basis for further optimization.

**Supplementary Information:**

The online version contains supplementary material available at 10.1186/s12934-022-01961-8.

## Background

Malic acid is a C4-dicarboxylic acid and essential intermediate of cell metabolism. While its main area of application is the food industry, it is also a constituent of personal care and cleaning products, pharmaceutical formulations, and a potential building block for the synthesis of polymers [1]. The global market demand for malic acid estimated at about 150,000 tons/year is currently mainly covered by chemical synthesis [2]. These processes use maleic anhydride as starting material which, in turn, is obtained via the catalytic vapor phase oxidation of hydrocarbons derived from fossil resources [1]. Microbial l-malic acid production cannot compare to chemical processes economically yet despite the availability of various natural and metabolically engineered production strains. Especially species of fungi such as *Ustilago*, *Rhizopus*, *Penicillium* and *Aspergillus* showed high yields and productivities [3]. In most of the high-performing malic acid production processes described so far, glucose was used as substrate. With this carbon source, l-malic acid concentrations of up to of 201 g/L, corresponding to a yield of 1.64 mol/mol and a productivity of 1.05 g/L/h, were reported using a modified strain of *A. niger* in a fed-batch process [4]. An even higher productivity was obtained with *A. oryzae* producing 1.38 g/L/h l-malic acid with a final concentration of 165 g/L and a yield of 0.91 mol/mol [5]. *A. oryzae* was also used in this study since it is a potent natural malic acid producer and accepts a broad substrate spectrum for the production of the organic acid [6].

The endeavor to establish a bio-based economy leads to the development of strategies for the optimum utilization of biomass. For this reason, and aiming for the reduction of process costs, side and waste streams from biomass processing such as lignocellulosic hydrolysates, thin stillage or crude glycerol are explored regarding their suitability as substrates for malic acid production [6–10]. We have previously evaluated acetate as carbon source for growth and organic acid production with *Aspergillus oryzae* [11, 12]. Acetate is a main component of the pyrolytic aqueous condensate, which is obtained as side stream during the pyrolysis of lignocellulosic biomass [13, 14]. It is also contained in lignocellulosic hydrolysates and can be produced through syngas fermentation [15–18]. Although l-malic acid production from acetate with *A. oryzae* is feasible, low yields and productivities were obtained compared to processes employing glucose. In a batch process, the applicable initial substrate concentration is limited due to inhibiting effects, resulting in a maximum l-malic acid concentration of about 8.4 g/L with 45 g/L acetic acid [11]. Due to acetate consumption, the pH strongly increases until inhibiting values are reached when uncontrolled. Furthermore, the cultivation of *A. oryzae* in media with a high acetate content resulted in the formation of a filamentous-lumpy morphology as opposed to defined biomass pellets which are obtained during cultivation on glucose. This filamentous morphology was linked to a low malic acid concentration of around 2 g/L when the fermentation was transferred to a bioreactor [12].

Therefore, the aim of this study was to improve the performance of l-malic acid production from acetate in 2.5-L bioreactor cultivations. This involved the development of a feeding and pH control strategy using acetic acid. An advantage accompanying the utilization of acetate is the possibility to control the pH solely by feeding the substrate in its protonated form. Furthermore, the influence of initial product concentrations of up to 50 g/L was investigated to evaluate a potential product inhibition.

## Results

### l-Malic acid production in 2.5-L bioreactor batch cultivations

At first, malic acid production with *A. oryzae* was evaluated in 2.5-L bioreactor batch fermentations. An overview of the differences between the fermentations presented in this study can be found in Table 1.

**Table 1:** Summary of cultivation conditions evaluated in 2.5-L bioreactors with a working volume of 1.4 L

Fermentation No.	CaCO_3_ [g/L]	pH correction
I	90	–
II	50	–
III	50	10 M acetic acid
IV	50	10 M acetic acid (pH 3.0)
V	50	10 M acetic acid (pH 4.5)
VI	90	10 M acetic acid
VII	90 + 42 g after 168 h	10 M acetic acid, 5 M NaOH

Stirrer velocity was 600 rpm, aeration 0.5 vvm and temperature 32 °C for all fermentations

**Fig. 1:**
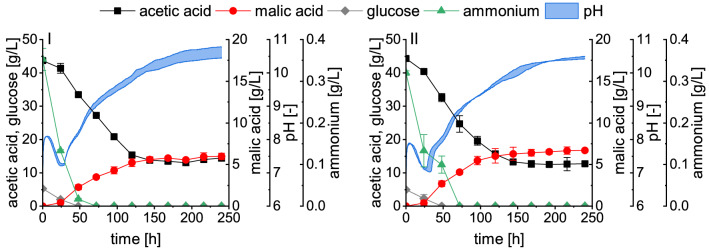
2.5-L bioreactor batch cultivations I (90 g/L CaCO_3_) and II (50 g/L CaCO_3_) without pH control. Fermentations were performed at 32 °C with a stirrer velocity of 600 rpm and an aeration rate of 0.5 vvm. Datapoints represent means ± standard deviation, n = 2

We have previously reported low malic acid production with acetate in 2.5-L bioreactor fermentations when using the same cultivation conditions as for glucose. While the malic acid yield with the saccharide in bioreactor fermentations was comparable to shake flask experiments, this was not the case using acetate. In these fermentations, a stirrer velocity of 300 rpm was used and only 0.73 ± 0.27 g/L malic acid was obtained within 144 h, representing less than 10% of the concentration obtained in shake flasks [12]. Increasing the stirrer velocity to 600 rpm (Fig. 1, I) considerably improved the fermentation outcome. Within 144 h, *A. oryzae* produced 5.62 ± 0.30 g/L malic acid, corresponding to a yield and productivity of 0.16 ± 0.01 g/g and 0.039 ± 0.002 g/L/h, respectively (Table 2). A distinct difference observed between the two stirrer velocities was the morphology of *A. oryzae*. During the bioreactor cultivation at 300 rpm, the filamentous-lumpy morphology obtained during growth in the seed culture was maintained and the biomass tended to attach to the bioreactor installations, limiting gas and nutrient transfer [12]. By increasing the agitation intensity to 600 rpm, spherical to elongated biomass pellets of about 1–2 mm in diameter were formed, enabling a better distribution of the biomass. The stirrer velocity is thus an important parameter for managing the morphology in acetate media and ensuring a successful scale-up.

**Table 2:** Fermentation results for 2.5-L bioreactor cultivations with *A. oryzae* for l-malic acid production

Fermentation No.	Substrate metabolized [g]^a^	Malic acid [g/L]	Y_P/S, malic acid_ [g/g]^b^	Malic acid productivity [g/L/h]	Total acid [g/L]^c^	Y_P/S, total acid_ [g/g]	Total acid productivity [g/L/h]	Sampling point for calculation [h]
I	50.12 ± 1.29	5.62 ± 0.30	0.16 ± 0.01	0.039 ± 0.002	11.99 ± 1.08	0.33 ± 0.04	0.083 ± 0.007	144
II	51.68 ± 0.74	6.29 ± 0.78	0.17 ± 0.02	0.044 ± 0.005	13.25 ± 1.47	0.35 ± 0.04	0.092 ± 0.010	144
III	104.47 ± 2.50	18.53 ± 0.90	0.26 ± 0.01	0.097 ± 0.005	35.82 ± 1.16	0.50 ± 0.00	0.187 ± 0.006	192
IV	96.62 ± 3.72	14.75 ± 1.20	0.23 ± 0.01	0.077 ± 0.006	28.90 ± 2.50	0.45 ± 0.01	0.151 ± 0.013	192
V	94.52 ± 2.78	16.22 ± 0.71	0.26 ± 0.00	0.084 ± 0.004	31.43 ± 1.02	0.50 ± 0.00	0.164 ± 0.005	192
VI	153.18 ± 6.12	29.53 ± 1.82	0.29 ± 0.01	0.112 ± 0.007	54.20 ± 3.23	0.53 ± 0.01	0.205 ± 0.012	264
VII	139.44 ± 6.60	18.66 ± 0.50	0.20 ± 0.01	0.071 ± 0.002	37.22 ± 2.49	0.40 ± 0.01	0.141 ± 0.009	264

Data represent means ± standard deviation, n = 2 (I–VI) or n = 3 (VII)

^a^Refers to the sum of substrate metabolized from acetate and glucose. Initially, 45 g/L acetic acid and 5 g/L of glucose were present in the medium

^b^Y_P/S, malic acid_ [g/g] = substrate specific malic acid yield calculated as g(malic acid)/g(consumed substrates)

^c^Refers to all organic acids produced during the cultivation (see Table 3)

Between 120 and 144 h the substrate consumption and malic acid production in fermentation I slowed down and ceased after 144 h. At this time point, the pH had exceeded a value of 10. This high pH seems to completely inhibit the metabolic activity of *A. oryzae*. During organic acid production with *A. oryzae* on glucose, CaCO_3_ is added as buffering agent which maintains the pH in a stable range between 6.5–7.0 without the further addition of an acid or base [12]. With glucose, the pH is mainly influenced by the consumption of ammonium and the production of organic acids, both leading to a decrease in pH. Using acetate, the consumption of the carbon source adds a further influencing factor. After a drop in pH during the consumption of ammonium and glucose at the beginning of fermentation, as shown in Fig. 1, the pH remains stable for a short time. This is followed by a phase of pH increase, which is accompanied by the consumption of acetate. When the metabolism is then inhibited due to a high pH, the pH slowly continues to increase, probably due to the loss of CO_2_ from the remaining CaCO_3_ through aeration. Compared to the experiments in shake flasks for which values close to pH 10 were only obtained after 240 h (Additional file 1: Fig. S1), the pH showed a faster increase in the bioreactor environment, leading to an inhibition. Therefore, complete substrate consumption was not observed. With about a third of the substrate remaining unused, lower maximum malic acid concentrations were obtained than in shake flask cultivations.

The presence of CaCO_3_ was shown to be beneficial for efficient malic acid production in shake flask cultivations [11]. However, the addition of CaCO_3_ also increases the pH value. For this reason, a lower CaCO_3_ concentration of 50 g/L was evaluated (Fig. 1, II) as opposed to the 90 g/L used in fermentation I. Even though the pH was slightly lower in the first part of the fermentation, inhibiting values were also reached between 120 and 144 h, resulting in the production of 6.29 ± 0.78 g/L malic acid and a yield of 0.17 ± 0.02 g/g within 144 h (Table 2). Hence, no considerable difference was observed between the two carbonate concentrations, even though malic acid production was correlated with the concentration of CaCO_3_ in shake flasks. Therefore, the lower concentration of 50 g/L was used for fermentations III-V. In these experiments, a pH-coupled feeding strategy was evaluated.

### l-Malic acid production in 2.5-L bioreactors using a pH-coupled feeding strategy

**Fig. 2:**
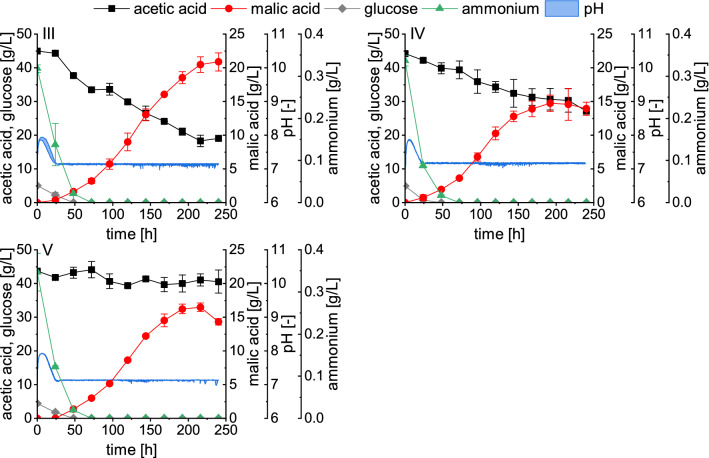
2.5-L bioreactor cultivations III-V with a pH-coupled feed and 50 g/L CaCO_3_. The pH was controlled with 10 M acetic acid (III), 10 M acetic acid pH 3.0 (IV), or 10 M acetic acid pH 4.5 (V). Fermentations were performed at 32 °C with a stirrer velocity of 600 rpm and an aeration rate of 0.5 vvm. Datapoints represent means ± standard deviation, n = 2

To prevent the pH from reaching an inhibiting range, acetic acid was used as pH controlling agent which simultaneously provided additional substrate during fermentation. As displayed for fermentations III-V in Fig. 2, the pH control and feeding of acetic acid started when the pH value reached its minimum, which was approximately after 24 h. During these first 24 h mainly glucose was consumed for growth and only a minor malic acid production was quantified. Glucose was added at a low concentration of 5 g/L to all bioreactor cultivations to reduce the lag phase and was depleted within 48 h in all fermentations.

In fermentation III (Fig. 2), the pH value was controlled with 10 M acetic acid. Compared to the batch fermentations displayed in Fig. 1, the period of malic acid production was extended to approximately 216 h. Within this time, 20.47 ± 1.17 g/L malic acid was produced which is more than three times as much as obtained in a batch process without pH control. After 216 h, malic acid production ceased. At this time point, the acetic acid concentration had decreased to a value of 18.34 ± 1.71 g/L. Hence, it was not possible to maintain the substrate concentration stable with this feeding strategy. We previously demonstrated that low initial acetate concentrations resulted in poor malic acid production and negatively affected the organic acid spectrum produced by *A. oryzae* [11]. The substrate concentration which is ideal to be maintained over an extended period, however, has not been determined yet. We therefore aimed to evaluate whether a constant substrate concentration close to the initial value of 45 g/L acetic acid would result in an increased maximum malic acid concentration and prolong the production time. One possibility to increase the amount of acetate added through the pH-coupled feed is to increase the pH value of the feeding solution. If the titrant is adjusted to a higher pH, a larger volume is required for pH correction, so more acetate is added to the fermentation broth. Therefore, fermentations with 10 M acetic acid set to a pH of 3.0 (Fig. 2, IV) or 4.5 (Fig. 2, V) as pH regulator were performed. When 10 M acetic acid with a pH of 3.0 was used, the substrate concentration also decreased during fermentation but to a lesser extent than with the unadjusted acid. After 216 h, 30.30 ± 3.50 g/L acetic acid were still available in the medium. In fermentation V, using acetic acid set to a pH of 4.5, the substrate concentration remained almost stable with a concentration of 41.07 ± 1.76 g/L determined after 216 h of fermentation. However, in both fermentations IV and V malic acid production ceased after about 192 h, thus earlier than in fermentation III when unadjusted acetic acid was used. Towards the end of these two fermentations, malic acid concentration decreased, indicating a reassimilation of the product. For comparison reasons, the calculations for fermentations III-V summarized in Table 2 are therefore based on the 192-h measurement point. Overall, fermentation III using the unadjusted 10 M acetic acid performed best, resulting in a malic acid concentration of 18.53 ± 0.90 g/L, a yield of 0.26 ± 0.01 g/g and a productivity of 0.097 ± 0.005 g/L/h. With acetic acid of pH 3.0, 14.75 ± 1.20 g/L malic acid was quantified and 16.22 ± 0.71 g/L using acetic acid with a pH of 4.5. Hence, maintaining the substrate concentration at a high level did neither increase the maximum malic acid concentration nor the timespan of acid production.

**Fig. 3:**
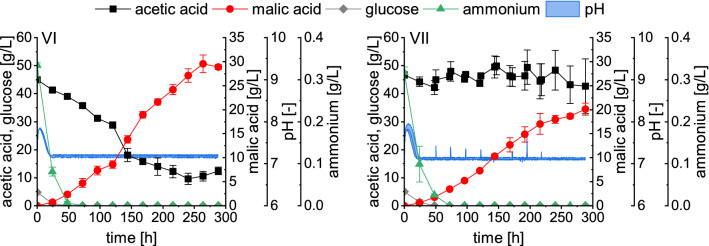
2.5-L bioreactor cultivations VI and VII with a pH-coupled feed and 90 g/L CaCO_3_. The pH was controlled with 10 M acetic acid (VI), or 10 M acetic acid and 5 M NaOH (VII). Fermentations were performed at 32 °C with a stirrer velocity of 600 rpm and an aeration rate of 0.5 vvm. Datapoints represent means ± standard deviation, n = 2 (VI) or n = 3 (VII)

In the later stages of fermentations III and IV a gradual dissolution of the CaCO_3_ was observed visually until depletion at around 216 h of cultivation. This might have contributed to the cessation of malic acid production despite carbon availability. Therefore, a higher initial concentration of 90 g/L CaCO_3_ was used again in fermentations VI and VII (Fig. 3). In fermentation VI, the pH and substrate concentration were regulated with 10 M acetic acid, whose pH was not adjusted. In fermentation VII, in addition to the 10 M acetic acid used for the pH-coupled feed, 5 M NaOH was manually added after each sampling between 48 and 216 h. Due to the addition of the base, more acetic acid was required for pH correction, resulting in higher substrate concentrations.

By increasing the CaCO_3_ concentration to 90 g/L and employing a pH-coupled feed consisting of 10 M acetic acid (fermentation VI) the highest malic acid concentration of 29.53 ± 1.82 g/L was obtained after 264 h (Table 2). This corresponds to a 5.3-fold increase compared to the batch process without pH control (fermentation I). The malate yield of 0.29 ± 0.01 g/g and the overall production rate of 0.112 ± 0.007 g/L/h were the highest of all processes. Malic acid productivity was maintained until about 264 h which was longer compared to the processes presented before. This suggests that a higher carbonate concentration has a positive effect on malic acid production with *A. oryzae* by extending the production time. During the 264 h, the acetic acid concentration decreased to 10.58 ± 1.69 g/L. Therefore, additional feeds of 5 M NaOH were used in fermentation VII. With these feeds, it was possible to maintain the acetic acid concentration above 40 g/L throughout the fermentation. To prevent CaCO_3_ depletion during this process, further 42 g CaCO_3_ was added after the sampling at 168 h. Despite high substrate concentrations and additional supply of CaCO_3_, malic acid yield and productivity were lower than in fermentation VI. This was comparable to the observations reported for fermentations IV and V. After 264 h, 18.66 ± 0.50 g/L malic acid were quantified, corresponding to a yield of 0.20 ± 0.01 g/g and a productivity of 0.071 ± 0.002 g/L/h.

Although malic acid was the main product of the cultivations presented in this work, *A. oryzae* secretes several other organic acids. With about 40% of the total acid production, succinic acid is the most significant side product, followed by fumarate with about 3–5% (Table 3). The pH-regulated feeding of acetic acid slightly increased the malate percentage and decreased the share of oxalate to below 1% compared to the batch fermentations. In the best performing fermentation VI, a total acid concentration of 54.20 ± 3.23 g/L, corresponding to a yield of 0.53 ± 0.01 g/g, was determined (Table 2). In this fermentation, malate production ceased after 264 h (Fig. 3). It is possible that product concentrations in this range inhibit further organic acid production of *A. oryzae*. The effect of initially added malate and succinate on the organic acid production was therefore evaluated in the following chapter.

**Table 3:** Organic acid distribution for bioreactor cultivations based on the sampling points summarized in Table 2

Fermentation No.	Organic acid distribution [%]^a^						
	Malate	Succinate	Fumarate	Pyruvate	α-Ketoglutarate	Oxalate	Citrate
I	47.0 ± 1.7	39.7 ± 2.4	5.3 ± 0.3	3.4 ± 0.7	0.3 ± 0.0	3.3 ± 0.1	1.1 ± 0.2
II	47.4 ± 0.6	40.7 ± 0.1	5.4 ± 0.1	2.3 ± 0.5	0.3 ± 0.0	3.0 ± 0.1	0.9 ± 0.0
III	51.7 ± 0.8	42.1 ± 0.5	3.8 ± 0.1	1.2 ± 0.2	0.2 ± 0.0	0.1 ± 0.0	1.0 ± 0.0
IV	51.0 ± 0.3	42.5 ± 0.4	4.2 ± 0.4	1.3 ± 0.0	0.1 ± 0.0	0.6 ± 0.2	0.3 ± 0.2
V	51.6 ± 0.6	42.0 ± 0.5	4.3 ± 0.0	1.5 ± 0.1	0.0 ± 0.0	0.6 ± 0.1	0.0 ± 0.0
VI	54.5 ± 0.1	40.2 ± 0.2	3.3 ± 0.2	0.6 ± 0.1	0.2 ± 0.0	0.9 ± 0.2	0.5 ± 0.1
VII	50.2 ± 2.7	43.1 ± 2.8	2.9 ± 0.2	1.6 ± 0.1	0.2 ± 0.0	0.8 ± 0.6	1.1 ± 0.1

^a^Values were calculated as the percentage of the total acid concentration as given in Table 2

### Organic acid production in the presence of malate and succinate

**Fig. 4:**
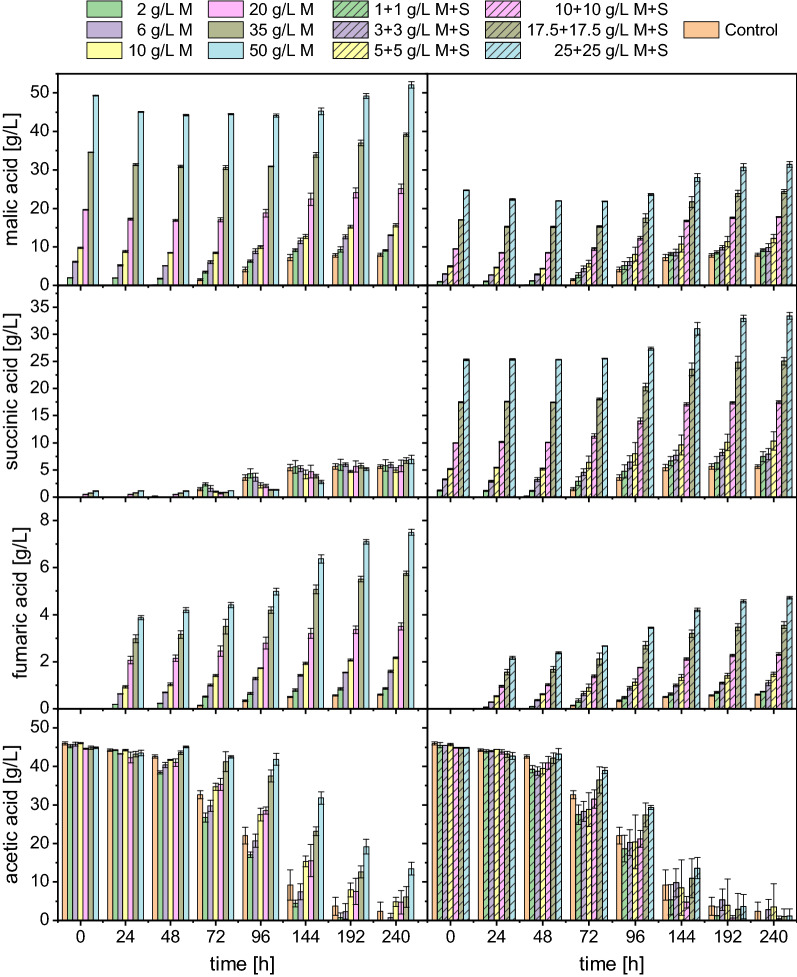
Organic acid production in shake flask cultivations with different initial malic and succinic acid concentrations. Malic acid was added at concentrations of 2–50 g/L (left column of figure) and combinations of malic and succinic acid were added at concentrations ranging from 1 + 1 g/L to 25 + 25 g/L (right column of figure). Cultures were incubated at 32 °C and 120 rpm using 90 g/L CaCO_3_. In the pre-culture, *A. oryzae* was grown using 45 g/L acetic acid as carbon source. *M*  malic acid, *S*  succinic acid. Datapoints represent means ± standard deviation, n = 3

High product concentrations can inhibit further organic acid production. Evaluating the influence of varying product concentrations can thus be interesting regarding the development of process strategies such as a repeated-batch fermentation with partial medium replacement. Therefore, the organic acid production of *A. oryzae* with medium containing up to 50 g/L malic acid was investigated. With about 40%, succinate makes up a large proportion of the totally produced organic acids for which reason combinations of malate and succinate were also tested. Figure 4 displays the production of malic, succinic and fumaric acid as well as the consumption of acetate determined for these shake flask cultivations. In these experiments, medium with 45 g/L acetic acid as carbon source was used which was supplemented with either 2–50 g/L malic acid (left column of Fig. 4) or combinations of malic and succinic acid at concentrations ranging from 1 + 1 g/L to 25 + 25 g/L (right column of Fig. 4). In the following, these values are referred to as “initial” product concentrations.

**Fig. 5:**
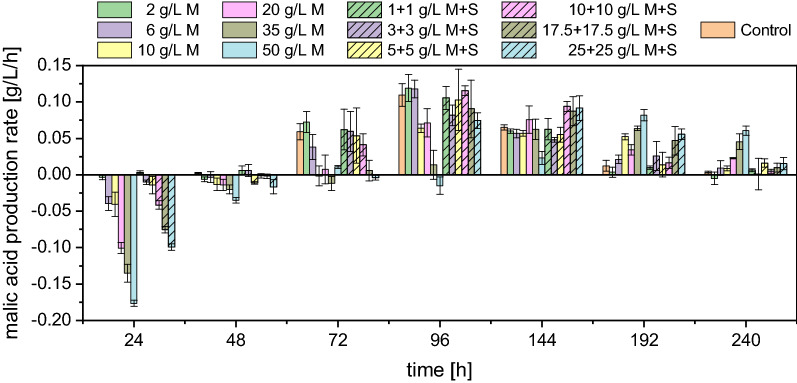
l-Malic acid production rates calculated between the individual sampling points. *M*  malic acid, *S*  succinic acid. Negative values represent a consumption. Datapoints represent means ± standard deviation, n = 3

As displayed in Fig. 4, all tested conditions first showed a decrease in the initial malic acid concentration before an increase was observed. The only exceptions were the control and the lowest combination of malic (M) and succinic acid (S), i.e., 1 + 1 g/L M + S. For the other conditions, the consumption of the initially available malate was between 10 and 20% before production of the acid started. This is also evident in Fig. 5, which shows the malic acid production rates calculated between the individual measurement points. Hence, the term “production” in this context refers to the increase in malic acid concentration after reaching the minimum concentration. This beginning of malic acid production after passing the minimum was generally observed later with a higher initial product concentration. In cultures with 35 g/L and 50 g/L initially added malic acid, considerable acid production was only detected after 144 h, illustrated by the calculated productivity which was only clearly in the positive range from this measurement point onwards (Fig. 5). The highest malic acid production rate was therefore detected later in these cultures. With 50 g/L malic acid, for example, the highest production rate of 0.082 ± 0.008 g/L/h was calculated between 144 and 192 h. The control and the cultures with a lower initial product concentration, on the other side, showed their maximum between 72 and 96 h which was in the range of 0.110–0.112 g/L/h. After 240 h a net production was determined for all tested conditions as all cultures had surpassed the initially added malic acid concentration. However, this net production was lower with a higher initial product concentration. The difference between the final malic acid concentration after 240 h and the concentration at the beginning, as well as the difference between the final and the minimal concentration, is displayed in Table 4. Regarding the difference between the final and the initial malate concentration, a net production of only 2.79 ± 0.75 g/L was determined with 50 g/L malic acid while 7.99 ± 0.42 g/L were measured for the control. When comparing the final minus the minimal concentration, however, a malic acid production of 8.02 ± 0.51 g/L was calculated for the cultures with 50 g/L malic acid which is comparable to the control. This shows that malic acid production is possible despite high product concentrations.

**Table 4:** Fermentation results for shake flask cultivations of *A. oryzae* with different initial product concentrations

Condition^a^	Acetic acid, initial-final^b^ [g/L]	Malic acid, final-initial [g/L]	Malic acid, final-minimal^c^ [g/L]	Y_P/S, malic acid, final-initial_ [g/g]	Productivity _malic acid, final-initial_ [g/L/h]	Succinic acid, final-initial [g/L]	Fumaric acid, final [g/L]	Total acid, final-initial [g/L]	Y_P/S, total acid, final-initial_ [g/g]	Productivity _total acid, final-initial_ [g/L/h]
2 g/L M	44.33 ± 0.38	7.18 ± 0.25	7.41 ± 0.12	0.16 ± 0.01	0.030 ± 0.001	5.75 ± 0.73	0.87 ± 0.03	14.34 ± 0.88	0.32 ± 0.02	0.060 ± 0.004
6 g/L M	44.18 ± 1.63	6.92 ± 0.11	7.98 ± 0.15	0.16 ± 0.00	0.029 ± 0.000	5.69 ± 0.43	1.59 ± 0.05	14.82 ± 0.37	0.34 ± 0.02	0.062 ± 0.002
10 g/L M	40.45 ± 1.28	5.88 ± 0.28	7.31 ± 0.26	0.15 ± 0.00	0.024 ± 0.001	4.92 ± 0.44	2.16 ± 0.03	13.90 ± 0.65	0.34 ± 0.01	0.058 ± 0.003
20 g/L M	39.87 ± 3.19	5.51 ± 1.19	8.37 ± 0.99	0.14 ± 0.02	0.023 ± 0.005	5.28 ± 1.06	3.50 ± 0.15	15.19 ± 1.86	0.38 ± 0.02	0.063 ± 0.008
35 g/L M	38.77 ± 3.10	4.56 ± 0.35	8.57 ± 0.37	0.12 ± 0.01	0.019 ± 0.001	5.98 ± 0.51	5.76 ± 0.09	16.90 ± 0.60	0.44 ± 0.02	0.070 ± 0.002
50 g/L M	31.43 ± 1.54	2.79 ± 0.75	8.02 ± 0.51	0.09 ± 0.02	0.012 ± 0.003	5.83 ± 0.82	7.49 ± 0.12	16.56 ± 1.58	0.53 ± 0.03	0.069 ± 0.007
1 + 1 g/L M + S	44.95 ± 0.09	8.23 ± 0.38	8.23 ± 0.38	0.18 ± 0.01	0.034 ± 0.002	6.36 ± 0.97	0.73 ± 0.01	15.78 ± 1.22	0.35 ± 0.03	0.066 ± 0.005
3 + 3 g/L M + S	41.87 ± 2.62	6.91 ± 1.00	7.18 ± 1.04	0.16 ± 0.01	0.029 ± 0.004	4.59 ± 0.94	1.09 ± 0.11	13.29 ± 1.82	0.32 ± 0.02	0.055 ± 0.008
5 + 5 g/L M + S	41.35 ± 6.19	7.24 ± 0.93	7.85 ± 1.17	0.18 ± 0.01	0.030 ± 0.004	5.14 ± 1.77	1.47 ± 0.10	14.49 ± 2.51	0.35 ± 0.01	0.060 ± 0.010
10 + 10 g/L M + S	44.40 ± 0.69	8.28 ± 0.11	9.32 ± 0.14	0.19 ± 0.00	0.035 ± 0.000	7.52 ± 0.25	2.32 ± 0.06	18.83 ± 0.38	0.42 ± 0.00	0.078 ± 0.002
17.5 + 17.5 g/L M + S	43.74 ± 2.00	7.36 ± 0.54	9.28 ± 0.42	0.17 ± 0.01	0.031 ± 0.002	7.59 ± 0.80	3.56 ± 0.15	19.48 ± 1.04	0.45 ± 0.01	0.081 ± 0.004
25 + 25 g/L M + S	43.77 ± 1.97	6.73 ± 0.69	9.60 ± 0.58	0.15 ± 0.01	0.028 ± 0.003	8.10 ± 0.64	4.71 ± 0.05	20.43 ± 1.15	0.47 ± 0.02	0.085 ± 0.005
Control	43.68 ± 2.80	7.99 ± 0.42	7.99 ± 0.42	0.18 ± 0.00	0.033 ± 0.002	5.63 ± 0.34	0.60 ± 0.02	15.03 ± 0.69	0.34 ± 0.01	0.063 ± 0.003

Data represent means ± standard deviation, n = 3

^a^*M* malic acid, *S* succinic acid

^b^Initial = acid concentration at 0 h; final = acid concentration at 240 h

^c^Minimal = minimal acid concentration. As displayed in Fig. 4, the malic acid concentration first showed a decrease from the initially added concentration before an increase was observed. The minimal concentration thus refers to these minimal values measured for each condition

The addition of succinate seems to have a lower impact on the organic acid production capacity of *A. oryzae* than malate. In the cultures with 20–50 g/L malic acid, low succinate concentrations were detected from the beginning of the cultivation which originated from impurities in the malic acid added to the medium. Different than malic acid, succinic acid was not consumed as no considerable decrease was observed even with the highest concentration of 25 + 25 g/L M + S. Most of the tested conditions with M + S showed a net malic acid production (final-initial) similar to the control (Table 4). Regarding the difference between the final and the minimal malate concentration, the cultures containing 10–25 g/L M + S even surpassed the control, with 25 + 25 g/L M + S producing 9.60 ± 0.58 g/L malate. This is an interesting observation as the acetate consumption was similar to the control. The net production of succinic acid over 240 h of cultivation was mostly between 5–6 g/L for all conditions and comparable to the control (5.63 ± 0.34 g/L). Only the cultures with 10–25 g/L M + S showed a higher production of up to 8.10 ± 0.64 g/L succinic acid.

Most of the initial malate consumption was observed within the first 24 h of cultivation. During the same period considerable amounts of fumaric acid were detected (Fig. 4) whereas fumaric acid was only detected after 72 h in the control cultures. The fumarate production was proportional to the initially added product concentration. Hence, the highest fumaric acid production of 3.88 ± 0.08 g/L after 24 h was determined for cultures with 50 g/L malic acid with a concurrent consumption of 4.24 ± 0.10 g/L malate. This corresponds to a molar production of 33.42 ± 0.73 mM fumaric acid and a consumption of 31.62 ± 0.75 mM malic acid. For the other tested conditions, the molar consumption and production were similar as well, and the proportion of malate converted to fumarate within the first 24 h of cultivation was between 9 and 13% (except for the control and 1 + 1 g/L M + S as no consumption was observed). This suggests that most of the initially consumed malate was converted to fumarate. In an abiotic control with medium incubated under the same conditions as the cultures with *A. oryzae*, no acid consumption or production was observed, clarifying that the fungal metabolism was involved in the production of fumaric acid (data not shown). After 240 h, the highest fumaric acid concentration of 7.49 ± 0.12 g/L was measured for cultures with 50 g/L malic acid compared to only 0.60 ± 0.02 g/L in the control cultures (Table 4). The addition of succinate had a lower impact on the production of fumaric acid since with the highest tested combination (25 + 25 g/L M + S) only about two thirds of the fumarate concentration obtained with 50 g/L malic acid, namely 4.71 ± 0.05 g/L, were quantified.

Besides the three main products malate, succinate and fumarate, only minor concentrations of other organic acids were detected. An overview of the organic acid spectrum for all tested conditions is listed in Table 5. The values in this table refer to the net acid production (final-initial concentration). The total acid concentration ranged from 13.29 ± 1.82 g/L (3 + 3 g/L M + S) to 20.43 ± 1.15 g/L (25 + 25 g/L M + S) (Table 4). The highest share of malate was quantified in the control (53.1 ± 0.5%), followed by succinate (37.4 ± 0.6%) and fumarate (4.0 ± 0.1%). The addition of malic and succinic acid mainly impacted the malate and fumarate proportions, leading to a decrease or increase, respectively. With 50 g/L malic acid, the malate share was reduced to 16.6 ± 3.0% while the fumarate portion was increased to 45.5 ± 4.2%.

**Table 5:** Organic acid distribution for shake flask cultivations of *A. oryzae* with different initial product concentrations

Condition^a^	Organic acid distribution [%]^b^						
	Malate	Succinate	Fumarate	Pyruvate	α-Ketoglutarate	Oxalate	Citrate
2 g/L M	50.1 ± 1.3	40.0 ± 2.7	6.1 ± 0.5	0.9 ± 0.1	0.3 ± 0.0	1.7 ± 0.5	0.9 ± 0.4
6 g/L M	46.7 ± 0.9	38.4 ± 1.9	10.8 ± 0.4	0.8 ± 0.0	0.5 ± 0.1	1.9 ± 0.5	1.0 ± 0.5
10 g/L M	42.3 ± 1.6	35.3 ± 1.5	15.6 ± 0.5	0.9 ± 0.1	0.5 ± 0.1	3.9 ± 0.7	1.5 ± 0.8
20 g/L M	36.0 ± 3.4	34.6 ± 2.6	23.3 ± 3.6	1.0 ± 0.2	0.4 ± 0.1	3.7 ± 2.2	1.0 ± 0.3
35 g/L M	26.9 ± 1.6	35.3 ± 1.9	34.1 ± 1.7	1.0 ± 0.1	0.3 ± 0.0	2.0 ± 0.7	0.3 ± 0.3
50 g/L M	16.6 ± 3.0	35.1 ± 1.6	45.5 ± 4.2	0.9 ± 0.1	0.3 ± 0.0	1.6 ± 0.5	0.0 ± 0.0
1 + 1 g/L M + S	52.2 ± 1.6	40.2 ± 3.0	4.6 ± 0.3	0.7 ± 0.0	0.3 ± 0.1	1.5 ± 0.8	0.4 ± 0.2
3 + 3 g/L M + S	51.9 ± 0.6	34.3 ± 2.4	8.3 ± 0.5	0.8 ± 0.2	0.4 ± 0.1	3.3 ± 2.3	1.0 ± 0.1
5 + 5 g/L M + S	50.3 ± 2.6	34.7 ± 6.8	10.3 ± 1.2	0.9 ± 0.1	0.5 ± 0.1	2.8 ± 2.7	0.7 ± 0.4
10 + 10 g/L M + S	44.0 ± 0.5	40.0 ± 0.6	12.3 ± 0.1	0.8 ± 0.1	0.4 ± 0.0	1.5 ± 0.3	1.0 ± 0.1
17.5 + 17.5 g/L M + S	37.8 ± 0.8	38.9 ± 2.0	18.3 ± 0.7	0.8 ± 0.1	0.3 ± 0.0	3.2 ± 2.4	0.7 ± 0.1
25 + 25 g/L M + S	32.9 ± 1.6	39.6 ± 0.9	23.1 ± 1.5	0.8 ± 0.1	0.3 ± 0.0	2.7 ± 0.7	0.5 ± 0.1
Control	53.1 ± 0.5	37.4 ± 0.6	4.0 ± 0.1	0.7 ± 0.0	0.5 ± 0.0	2.8 ± 1.0	1.4 ± 0.2

^a^*M*  malic acid, *S*  succinic acid

^b^Values refer to the organic acid distribution measured at 240 h minus the initial concentrations of malic and succinic acid and are given as percentage of the total acid concentration which is displayed in Table 4

Data represent means ± standard deviation, n = 3

Regarding the acetate consumption, the substrate was depleted or close to depletion in most cultures after 240 h except for those containing higher malate concentrations. In the cultures with 50 g/L initial malic acid, 13.4 ± 1.7 g/L acetic acid were left at this measurement point, suggesting that malate production could have continued even further. Considering the delayed consumption of acetate and the nitrogen source ammonium (see Additional file 1: Fig. S1 which also displays the development of the pH), it is likely that high product concentrations inhibit the growth of *A. oryzae*. This inhibition, however, seems to originate from malic acid primarily as the effect of the combination of malate and succinate was less pronounced.

## Discussion

### l-Malic acid production in 2.5-L bioreactor batch cultivations

This work aimed to improve malic acid production with *A. oryzae* using acetate as carbon source in 2.5-L bioreactor fermentations. At first, organic acid production was evaluated in batch processes (Fig. 1). Compared to previously reported results, malate production was increased considerably by applying a stirrer velocity of 600 rpm instead of 300 rpm [12]. The increase in agitation velocity induced a change in morphology from filamentous to pelleted which likely was the main reason for the improved acid production. The morphology developed by filamentous microorganisms can take a variety of different forms from dispersed hyphae to compact pellets. Thereby, the ideal morphology seems to vary between target products and microorganisms [19]. The pellet formation is influenced by various factors such as the inoculum concentration, osmolality, pH, aeration rate, stirrer velocity, and the presence of additives including surfactants and particles [19, 20]. In the cultivations presented in this work, biomass pellets were formed only during the fermentation in the bioreactor, not in the seed culture in which a filamentous-lumpy morphology was obtained. Thus, the different cultivation conditions in the bioreactor, likely the higher shear rate in particular, caused this transition. A high stirrer velocity usually results in the formation of smaller and more compact biomass pellets due to the breaking of pellets or the erosion of the pellet surface [21–23]. However, comparing low agitation intensities, it seems that the pellet diameter first increases before high shear forces lead to a decrease. For *Trichoderma reesei* a transition towards larger and more defined pellets was observed when increasing the stirrer velocity from 100 to 400 rpm in 7.5-L bioreactor cultivations [24]. Similar observations were reported for the cultivation of *A. oryzae* FCD15 in 7.5-L bioreactors for malate production. When the stirrer velocity was increased from 200 to 600 rpm, the morphology changed from a mycelial to a pelleted form and the malate concentration after 144 h increased from approximately 40 g/L to 83.3 g/L [25]. Further experiments should evaluate the influence of the stirrer velocity on morphology and organic acid production with *A. oryzae* from acetate in more detail to find the optimum balance between malate production and energy demands.

### l-Malic acid production in 2.5-L bioreactors using a pH-coupled feeding strategy

In the bioreactor batch processes, malic acid production ceased after 144 h despite carbon availability which was most likely a result of the high and inhibiting pH values. Using a pH-coupled feed of acetic acid, the pH was maintained at physiological values of about 7.1–7.2 and additional substrate was provided at the same time. With a feed of 10 M acetic acid and a CaCO_3_ concentration of 90 g/L (fermentation VI, Fig. 3), the maximum malic acid titer was increased about 5.3-fold compared to the batch process without pH control, yielding 29.53 ± 1.82 g/L. This is the highest malic acid concentration obtained with acetate as the main carbon source so far. The amount of substrate provided through the pH-coupled feed, however, was not sufficient to maintain a stable acetate concentration. With an acetic acid feed adjusted to a pH of 4.5 (fermentation V), and by applying manual feeds of 5 M NaOH (fermentation VII), the acetate concentration was successfully maintained close to the initial concentration of 45 g/L acetic acid. These strategies, however, resulted in lower malic acid concentrations compared to the fermentations using unadjusted 10 M acetic acid only. A reason for this observation might be a high ion concentration. To adjust the pH of the 10 M acetic acid with a pH of 4.5, sodium hydroxide was used. Due to the pH-dependent feeding of acetic acid with pH 4.5, or the manual feeds of 5 M NaOH, sodium ions accumulated during the fermentation. It is possible that these additional ions inhibited malic acid production in later stages of the cultivation. Inhibition of production caused by sodium chloride concentrations of 40 g/L (0.68 mol/L) and above was also observed during Ochratoxin A synthesis with *Aspergillus ochraceus* and *Penicillium nordicum* [26]. Another factor which might have contributed to the decreased malic acid production in fermentations IV, V and VII is a higher concentration of acetic acid in the medium. It is assumed that the growth inhibiting effect of acetate is caused by the presence of its protonated form which can enter the cells by diffusion, causing an acidification of the cytoplasm upon deprotonation [27]. In the fermentations with pH control through 10 M unadjusted acetic acid (III and VI), the acetate concentration decreased more rapidly, leading to a lower concentration of the protonated form in the fermentation medium. When a higher acetate concentration was maintained, *A. oryzae* was subjected to elevated concentrations of the protonated form for a longer period. Even though the ratio of acetic acid to acetate at a pH close to neutrality is low (more than 99% is deprotonated at pH 7.0), a prolonged subjection may be inhibiting and contribute to a lower productivity. In further experiments, the feeding strategy should be optimized and the effect of maintaining lower substrate concentrations on malic acid production should be evaluated. Additionally, the optimum pH value for organic acid production with *A. oryzae* in acetate media should be determined. The utilization of acetic acid for substrate supply and pH control has also been described for other processes and different regulation strategies were employed. For lipid production with *Rhodotorula glutinis*, the pH was maintained using 30% (v/v) acetic acid. In the reported process, this could keep the acetate supply at a sufficiently high level with only little fluctuations around the initial concentration [28]. Qian et al. employed the same strategy for lipid production with the yeasts *Cryptococcus podzolicus* and *Trichosporon porosum* using 4 M acetic acid for pH control and substrate supply [29]. For lipid production with *Yarrowia lipolytica*, it was reported that utilizing acetic acid for pH control alone was not sufficient to provide an adequate substrate supply. The authors therefore added a continuous sodium acetate feed to maintain a low acetate concentration throughout the semicontinuous fermentation [30]. Another strategy combines the pH-coupled feeding of acetic acid with feeds of an acetate solution depending on the dissolved oxygen concentration. This was employed for itaconic acid production with *Corynebacterium glutamicum.* When the DO-concentration surpassed an upper limit upon the depletion of the carbon source, acetate solution was fed which allowed for the production of 29.2 g/L itaconate within 46 h [31]. Maintaining a low acetate concentration with a continuous or DO-coupled acetate feed could also be evaluated for malic acid production with *A. oryzae*.

Interestingly, malic acid production ceased despite the availability of residual acetate in fermentations III-VI, followed by a reassimilation in fermentations IV and V. This was also observed in batch processes with *A. oryzae* using glucose as substrate, reporting malate reassimilation despite a residual glucose concentration of about 30 g/L [32]. In this regard, optimizing the supply of medium components such as the nitrogen availability or salt concentration could be advantageous. While nitrogen limitation is important for an efficient organic acid synthesis in filamentous fungi, the C/N ratio can influence the organic acid composition [33, 34]. Thus, an adequate supply of nitrogen can positively influence malic acid production. Ji et al. showed that an optimized nitrogen supply strategy could result in an prolonged phase of rapid malate production with *A. oryzae*, leading to an increase in product concentration from 130.5 to 164.9 g/L [35]. Regarding malate production with *Ustilago trichophora*, increasing the NH_4_Cl concentration enhanced the maximum volumetric productivity and therefore enabled a higher malate titer in a shorter time [8]. Schmitt et al. did not observe a positive effect of supplementing the glucose feed with nitrogen in fed-batch processes with *A. oryzae* but found an increased malate concentration when complete medium was added [32]. Maybe, the C/N ratio in these experiments was not ideal. In a study with *A. oryzae* FMME218-37, the C/N ratio was found to have a significant impact on the maximum malate titer. At a fixed C/N ratio, however, also the concentrations of the carbon and nitrogen source were shown to influence malic acid production. With a C/N ratio of 50 and optimized glucose and ammonium sulfate concentrations of 146 g/L and 5.5 g/L, respectively, a high malate titer of 88.4 g/L was reported [36]. However, an increased availability of nitrogen enhances biomass formation, which diverts the carbon from malate synthesis. The nitrogen availability must thus be balanced between productivity and yield. Optimizing the availability of nitrogen and other medium components could be a tool to extend the production period and increase the productivity for malate synthesis from acetate and should be evaluated in further studies.

Two concentrations of calcium carbonate were evaluated both in batch mode (fermentations I and II) and in cultivations with a pH-coupled feed (fermentations III and VI). While malic acid production with the tested carbonate concentrations was similar in batch fermentations, the utilization of 90 g/L CaCO_3_ resulted in an increased malate titer in cultivations with a pH-coupled feed as carbonate depletion was prevented. Hence, an adequate supply of CaCO_3_ throughout the fermentation seems necessary for efficient production of malic acid. CaCO_3_ can influence the cultivation in several ways. It neutralizes the produced organic acids, provides insoluble solids, and is a source for calcium ions and CO_2_. The availability of carbon dioxide potentially plays a role in malate production from glucose as pyruvate carboxylase fixes CO_2_ in the reductive tricarboxylic acid cycle, which is assumed to be the main pathway towards malate production in *Aspergillus* using this substrate [37]. Furthermore, the provided Ca^2+^ can form poorly soluble calcium malate which precipitates and helps prevent product inhibition [8]. CaCO_3_ is therefore often used in processes for organic acid production. In cultivations with *A. oryzae* performed by Geyer et al., a high CaCO_3_ concentration of 120 g/L was reported advantageous as an increased carbon percentage was attributed to malic acid [38]. The concentration of CaCO_3_ was also found to affect organic acid production by *Rhizopus delemar*. With 60 or 100 g/L CaCO_3_ compared to 20 g/L, carbonate was available until the end of the fermentation, which prevented a drop in pH and resulted in a higher malic to fumaric acid ratio. The authors ruled out the presence of insoluble solids as influencing factor but found increased ion concentrations and different pH values to affect the production profile [39]. Schmitt et al. also observed a positive effect of calcium carbonate on malic acid productivity and associated it with its ability to control the pH as buffering agent, although the authors pointed out that this may not be its only function [32]. A high carbonate concentration is accompanied by a high ion concentration which remains in the medium once the carbonate is exhausted and might as well become inhibiting. With a concentration of 50 or 90 g/L CaCO_3_, 20 or 36 g/L Ca^2+^ were provided, respectively. Ronoh et al. reported a decreased glucose consumption and fumaric acid production of *R. delemar* with 20 g/L Ca^2+^ in the medium, which suggests inhibition. However, they also observed an increased, albeit delayed, formation of malic acid which might be attributed to a stimulatory effect of Ca^2+^ on the enzyme fumarase [39]. The results described in all these references were obtained for cultivations using glucose as carbon source. So far, the function of CaCO_3_ in malic acid production from acetate could not be identified conclusively and should be the subject of future studies. Furthermore, a potential inhibition of *A. oryzae* by calcium ions needs to be evaluated. Considering a possible inhibition by both the calcium ions provided through the CaCO_3_ and the sodium ions added through an acetate feed required for maintaining a stable substrate concentration, a fermentation strategy with partly removal of the fermentation medium will probably be necessary to maintain a high production rate.

Compared to the maximum malic acid productivities and yields reported with glucose, the values obtained with acetate presented in this study are considerably lower. High productivities and yields were for example determined by Ji et al. (1.14 g/L/h, 0.77 g/g), Chen et al. (1.08 g/L/h, 0.75 g/g), Liu et al. (1.38 g/L/h, 0.68 g/g) and Xu et al. (1.05 g/L/h, 1.22 g/g) for different species of *Aspergillus* [4, 5, 25, 35]. These strains, however, were all genetically modified. Given the high production of side products, especially succinate, with acetate as carbon source, a possibility for increasing the malate yield is the reduction of these organic acids by directing the carbon flux towards malic acid. Acetate is metabolized through the glyoxylate pathway. In this bypass of the citric acid cycle, isocitrate is first converted to glyoxylate and succinate by the enzyme isocitrate lyase, followed by a conversion of the glyoxylate and acetyl-CoA to malate by malate synthase. The use of this pathway for acetate metabolization in *Aspergillus* was demonstrated in cultivations with *A. nidulans*, where induction of the two enzymes was observed in the presence of acetate. Furthermore, mutants deficient in either enzyme were not able to grow on this carbon source [40, 41]. Experiments with 13C-labeled acetate demonstrated the incorporation of the labeled carbon into citrate, malate and succinate in *A. fumigatus* [42]. The production of high succinate concentrations is thus a result of the glyoxylate cycle as main pathway towards malic acid production. Liu et al. found that the succinate concentration could be decreased in favor of an enhanced malate titer in *A. oryzae* by two strategies. As the authors found that succinic acid was mainly present in the cytosol, they overexpressed a dicarboxylate carrier from *Saccharomyces cerevisiae* which transports succinate from the cytosol to the mitochondria. Furthermore, they engineered the redox metabolism by overexpression of a water-forming NADH oxidase [43]. As the succinic acid proportion obtained during malic acid production from acetate represents approximately 40% of the total acids produced, there is a large potential for improvement which should be evaluated in further studies.

### Organic acid production in the presence of malate and succinate

The observed cessation of malic acid production in later stages of the fermentation could also be caused by product inhibition. Therefore, the effect of malate and succinate addition on organic acid production was evaluated in shake flask cultivations. Interestingly, a high fumarate production was observed especially in the first 24 h of the cultivation with a concurrent consumption of malate. This fumarate production was proportional to the amount of malic acid added while the addition of succinate only had a minor effect. Fumarase, which was shown to be present both in the mitochondria and the cytosol in *Aspergillus* species, catalyzes the reversible hydration of fumaric to l-malic acid [37]. Experiments with purified porcine fumarase and permeabilized yeast cells showed that the maximal enzymatic activity to fumaric acid is considerably higher than to malic acid and that about 80% of fumarate are converted to malate under equilibrium conditions [44]. The high fumarate production observed at high product concentrations is likely a result of the dehydration of malate to fumarate. Interestingly, in fermentation VI, the fumarate proportion amounted to only 3.3 ± 0.2% total acid despite the presence of almost 30 g/L malic acid. In the bioreactor processes, however, high malate concentrations were obtained only in later stages of the fermentation when the cultivation conditions such as the pH and nutrient concentrations were different than in shake flasks. The pH value was shown to influence the direction of the fumarase-catalyzed reaction as the maximum activity towards fumarate hydration was pH 7, while it was about pH 8 for the dehydration using porcine fumarase [44]. The different metabolic states of *A. oryzae* or the C/N ratio may also have played a role in fumarate production. The steepest increase in fumarate concentration in shake flasks was observed within the first 24 h. During this time, *A. oryzae* was still in the lag phase as only minor differences in the ammonium concentration were observed (Additional file 1: Fig. S1). In the bioreactor fermentations, however, nitrogen was depleted between 48 and 72 h and the growth phase therefore completed when high malic acid titers were present. It is also conceivable that the transport of malic acid out of the cell during the production phase is rapid, preventing dehydration to fumaric acid. For a better understanding of the fumarate production observed at high product concentrations, fumarase activity in *A. oryzae* needs to be further evaluated. These experiments should ideally be performed in the controlled environment of a bioreactor with pH control.

Another interesting observation of the experiments with malate and succinate addition is the total acid production in relation to the consumed substrate (Table 4). In the control, the total acid production was 15.03 ± 0.69 g/L. Especially the addition of succinate increased the total acid concentration, since the highest titer of 20.43 ± 1.15 g/L was measured with 25 + 25 g/L M + S. The substrate consumption in these cultures, however, was similar. The reassimilation of malate or succinate, or the conversion of these compounds into other intermediates or products such as fumarate, might be more favorable regarding the demand for energy or reduction equivalents. It is also possible that the biomass formation was influenced by the availability of the two compounds which then affected the production capacity. The biomass concentration was not measured in this work but considering the ammonium measurements, fungal growth was delayed with increasing concentration of succinate and especially malate. This delay in ammonium consumption might as well originate from an inhibition caused by a higher sodium ion concentration as more sodium hydroxide was required for pH correction in these media and not necessarily from the products themselves. Further research is therefore required to clarify these observations.

The question whether product concentrations of up to 50 g/L inhibit malate production cannot be answered conclusively by the results presented here. Even though product formation was delayed, and lower maximum productivities were obtained with high initial product concentrations, malic acid production was still observed. Given that the differences between the final and the minimal malic acid concentrations were comparable or, in the case of 10–25 g/L M + S, even higher than in the control, an inhibition regarding the organic acid production capacity in a range up to 50 g/L seems not to be very pronounced. Evaluating the effects of high malate and succinate concentrations for selected key enzymes involved in malate production from acetate individually could be a tool to elucidate a possible product inhibition in more detail. Furthermore, adding the products after the depletion of the nitrogen source rather than at the beginning of cultivation should be considered.

## Conclusions

This work evaluated several strategies to improve malic acid production using acetate as carbon source in 2.5-L bioreactor fermentations with *A. oryzae*. Using a higher stirrer velocity induced the formation of biomass pellets which seemed crucial for a successful scale-up from shake flask to bioreactor. With a pH-coupled feeding strategy, the malic acid concentration was increased, resulting in a maximum concentration of 29.53 ± 1.82 g/L which is the highest concentration obtained with acetate as the main carbon source so far. It was found that the pH-coupled feed of unadjusted acetic acid was not sufficient to provide a constant substrate concentration. Maintaining the substrate supply within a constant range close to the initial concentration of 45 g/L acetic acid, however, did not prove beneficial. Shake flask experiments to evaluate the effects of high product titers showed a stronger influence of malate than of combinations of malate and succinate. Despite a delay in growth and acid secretion observed with high product concentrations, malate production was similar to the control after an initial reassimilation, illustrating that product synthesis with *A. oryzae* beyond 50 g/L is possible in acetate medium. In further experiments the feeding strategy should be optimized with the aim to prolong the period of maximum productivity. Aspects to consider could be a feed of medium components such as salts and nitrogen and maintaining a lower acetate concentration throughout the fermentation. Since succinic acid accounts for about 40% of the total acid production, strategies such as metabolic engineering aiming to decrease the concentration of this side product should be considered to maximize malic acid yield.

## Material and methods

### Microorganism and media

*Aspergillus oryzae* DSM 1863 was obtained from DSMZ strain collection (Deutsche Sammlung von Mikroorganismen und Zellkulturen GmbH, Braunschweig, Germany) and spore propagation was performed as described previously [12].

Organic acid production was carried out as a two-stage process consisting of a pre-culture and a main culture. The pre-culture medium consisted of 45 g/L acetic acid, 4 g/L (NH_4_)_2_SO_4_, 0.75 g/L KH_2_PO_4_, 0.98 g/L K_2_HPO_4_, 0.1 g/L MgSO_4_·7H_2_O, 0.1 g/L CaCl_2_·2H_2_O, 5 mg/L NaCl, 5 mg/L FeSO_4_·7H_2_O and 2 mL/L 1000 × Hutner’s Trace Element solution. 1000 × Hutner’s trace element solution consisted of 5 g/L FeSO_4_·7H_2_O, 50 g/L EDTA-Na_2_, 22 g/L ZnSO_4_·7H_2_O, 11 g/L H_3_BO_3_, 5 g/L MnCl_2_·4H_2_O, 1.6 g/L CoCl_2_·6H_2_O, 1.6 g/L CuSO_4_·5H_2_O, and 1.1 g/L (NH_4_)_6_Mo_7_O_24_·4H_2_O with a pH of 6.5 [45]. The pH of the pre-culture medium was set to 6.5 with NaOH.

The main culture medium for organic acid production in 2.5-L bioreactors was composed of 45 g/L acetic acid, 1.2 g/L (NH_4_)_2_SO_4_, 0.1 g/L KH_2_PO_4_, 0.17 g/L K_2_HPO_4_, 0.1 g/L MgSO_4_·7H_2_O, 0.1 g/L CaCl_2_·2H_2_O, 5 mg/L NaCl, 60 mg/L FeSO_4_·7H_2_O, and 50 or 90 g/L CaCO_3_. Furthermore, 5.0 g/L glucose were added to the production medium in the bioreactor fermentations in form of a stock solution (c = 400 g/L) sterilely after autoclaving to shorten the lag phase. The main culture media for evaluating the influence of different product concentrations on acid production in shake flask cultivations had the same composition. The only differences were that no glucose was added to these media, that 90 g/L CaCO_3_ was used for all tested conditions, and that malic and succinic acid in concentrations of 1–50 g/L were added as indicated in the results section. The pH of the main culture media was adjusted to a value of 5.5 with NaOH. All media were sterilized by autoclaving for 20 min at 121 °C.

### Pre-culture conditions

In 500-mL shake flasks, 100 mL of the pre-culture medium were inoculated with 3 × 10^7^ conidia and incubated at 30 °C and 100 rpm. After 48 h, the biomass was separated from the medium by filtration through Miracloth (Merck KGaA, Darmstadt, Germany), washed thoroughly with distilled water and resuspended in main culture medium. For the bioreactor cultivations, the biomass of four pre-cultures was resuspended in 150 mL of main culture medium which was used for the inoculation of one bioreactor. For the shake flask cultivations, the biomass of two pre-cultures was resuspended in 200 mL of the respective main culture medium and 10 mL were used for the inoculation of one shake flask.

### Organic acid production in bioreactors

Organic acid production was performed in 2.5-L stirred tank reactors (Minifors, Infors HT, Bottmingen, Switzerland) with a working volume of 1.4 L. 0.3 mL of the antifoaming agent Contraspum A 4050 HAc (Zschimmer und Schwarz GmbH und Co KG, Lahnstein, Germany) was added before inoculation and when necessary during the fermentation. The agitator consisted of two 6-bladed Rushton turbine impellers with a diameter of 4.6 cm and a blade width of 1.1 cm. The distance between the two impellers was 8.0 cm. The cultivations were carried out at 32 °C, 600 rpm and an aeration rate of 0.5 vvm. In Table 1 an overview is given of the different cultivation conditions. Fermentations were run either without pH adjustment (fermentations I and II) or with an automatic pH control using 10 M acetic acid (fermentations III–VII). The pH control was activated when the pH value reached its minimum after an initial peak, which was approximately after 24 h of cultivation. The target pH was set to this minimum value measured online which was between pH 7.1–7.2. For the pH-adjustment to pH 3.0 and 4.5 of the 10 M acetic acid used in fermentations IV and V solid NaOH was used. In fermentation VII, besides the pH-coupled feed of 10 M acetic acid, manual feeds of 5–10 mL of 5 M NaOH were performed after each sampling between 48 and 216 h to increase the amount of acetic acid required for pH control. The volume of the NaOH feed for each bioreactor was determined after HPLC measurement of the acetic acid concentration in the fermentation broth. The 5 M NaOH was added over a period of several minutes to prevent the pH from increasing to potentially inhibiting values. Additionally, 42 g of CaCO_3_ (one third of the initial amount) was added after the sampling at 168 h during fermentation VII to prevent carbonate depletion. Cultivations were run in duplicate except for fermentation VII for which a triplicate was performed. The pH value was measured online. Samples were taken at the indicated time points for offline analysis of ammonium, glucose and organic acid concentration.

### Organic acid production in shake flasks

In 500-mL shake flasks, 100 mL of the main culture medium were inoculated with 10 mL resuspended pre-culture and incubated at 32 °C and 120 rpm for 240 h. Samples were taken at the indicated time points for the determination of pH as well as organic acid and ammonium concentration. All experiments were performed as triplicates.

### Analytics

Organic acids and glucose were quantified by HPLC (Agilent 1100 Series) using a Rezex ROA organic acid H + (8%) column (300 × 7.8 mm, 8 µm, Phenomenex) with a UV detector at 220 nm for organic acid and an RI detector for glucose determination. Sample preparation and conditions for analysis are described elsewhere [12].

Ammonium was measured photometrically (Spectroquant Test Kit, 114,752, Merck KGaA). The reaction volume of the assay was scaled down to 200 µL and samples were measured in duplicate in microtiter plates according to the instructions of the manufacturer.

## Supplementary Information

**Additional file 1: Figure S1.** Ammonium consumption and pH development during shake flask cultivation of *A. oryzae* with different initial malic (M) and succinic acid (S) concentrations. Datapoints represent means ± standard deviation, n = 3.

## Data Availability

The datasets supporting the conclusions of this article are included within the article and its additional file.
