# Supplementary material for: Optimization of l-malic acid production from acetate with Aspergillus oryzae DSM 1863 using a pH-coupled feeding strategy
Source: Microb Cell Fact. 2022 Nov 23;21:242. doi: 10.1186/s12934-022-01961-8 (PMC9685910; doi:10.1186/s12934-022-01961-8)
Supplement: Supplementary file 1 — Additional file 1: Figure S1. Ammonium consumption and pH development during shake flask cultivation of A. oryzae with different initial malic (M) and succinic acid (S) concentrations. Datapoints represent means ± standard deviation, n = 3. [file 12934_2022_1961_MOESM1_ESM.pdf]

Additional information

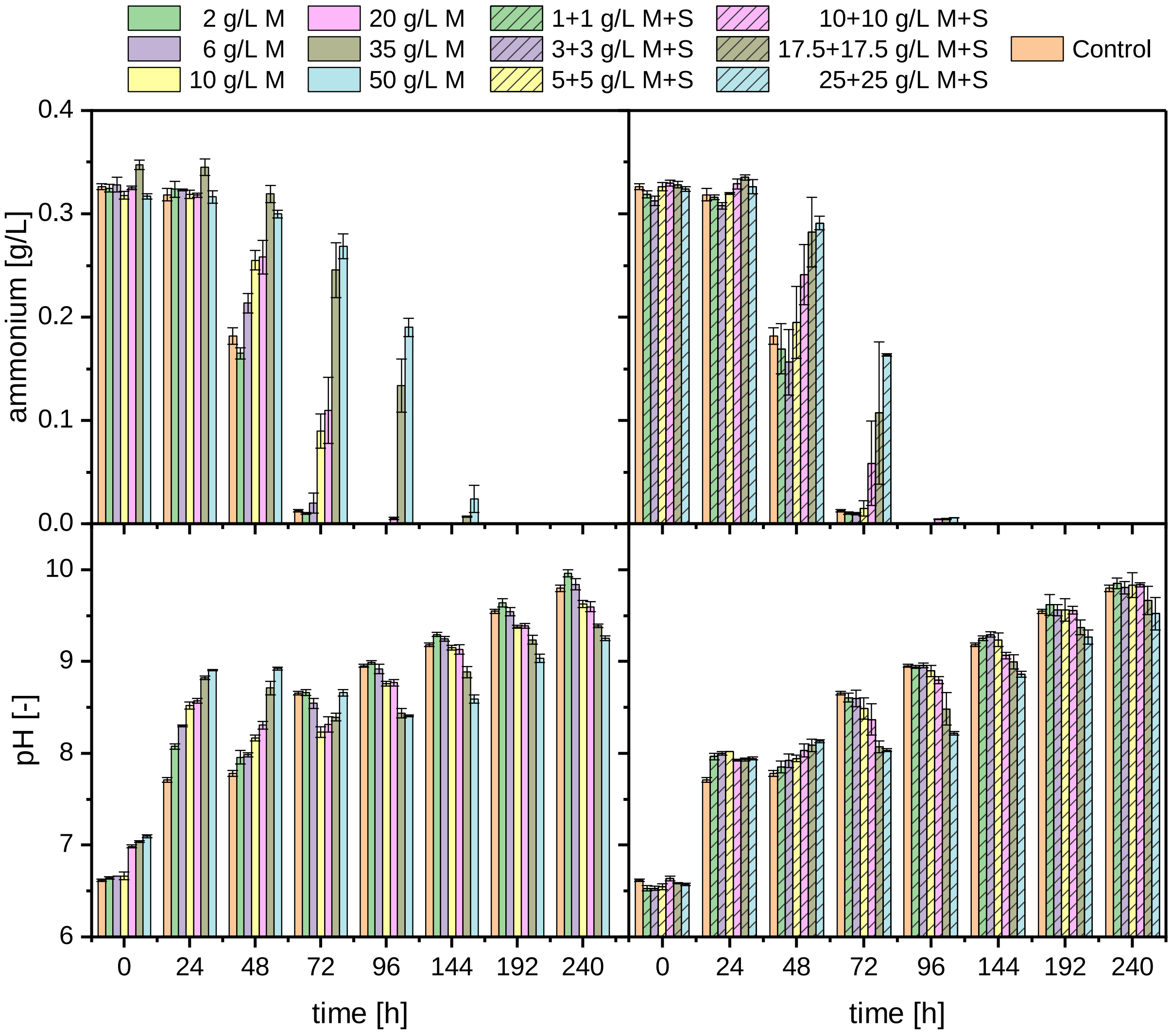

Figure S1. Ammonium consumption and pH development during shake flask cultivation of *A. oryzae* with different initial malic (M) and succinic acid (S) concentrations. Datapoints represent means ± standard deviation, n = 3.
